# Osteological correlates of the respiratory and vascular systems in the neural canals of Mesozoic ornithurines *Ichthyornis* and *Janavis*

**DOI:** 10.1002/ar.70070

**Published:** 2025-10-09

**Authors:** Jessie Atterholt, M. Grace Burton, Mathew J. Wedel, Juan Benito, Ellen Fricano, Daniel J. Field

**Affiliations:** ^1^ College of Osteopathic Medicine of the Pacific Western University of Health Sciences Pomona California USA; ^2^ Department of Earth Sciences University of Cambridge Cambridge UK; ^3^ College of Podiatric Medicine Western University of Health Sciences Pomona California USA; ^4^ Museum of Zoology University of Cambridge Cambridge UK

**Keywords:** mesozoic, neural canal, ornithurine, pneumatic foramina, soft‐tissue anatomy, vascular foramina

## Abstract

In birds, the neural canal houses a variety of anatomical structures including the spinal cord, meninges, spinal vasculature, and respiratory diverticula. Among these, paramedullary diverticula and the extradural dorsal spinal vein may leave behind osteological correlates in the form of pneumatic foramina and fossae, and a bilobed geometry of the neural canal, respectively. While recent studies have cast light on the evolution of avian skeletal pneumaticity, evidence for these respiratory and vascular structures has never been reported in Mesozoic ornithurines, raising questions about the evolutionary origins of these modern components of the avian respiratory and vascular systems. Here, we investigated the neural canals of *Ichthyornis* and *Janavis*, which provide the first evidence of paramedullary diverticula and spinal vasculature in Mesozoic ornithurine birds. In both taxa, numerous pneumatic foramina are present inside the vertebral canals, primarily in the cervical and thoracic vertebrae. *Ichthyornis* and *Janavis* also both exhibit evidence of a large, extradural dorsal spinal vein in the cervical and thoracic regions, as indicated by a bilobed geometry of the neural canal. Some vertebrae of *Ichthyornis* also preserve paired ventrolateral channels suggesting the presence of additional spinal vessels, although a lack of information on spinal vasculature in extant birds hinders identification of specific vascular structures. These results cast new light on the detailed soft tissue anatomy of *Ichthyornis* and *Janavis*, and affirm the utility of these osteological correlates which can be applied to other fossil avialans.

## 1 INTRODUCTION

The neural canal of vertebrates houses and protects the spinal cord, a structure that exhibits little morphological variability through tetrapod evolutionary history (Butler & Hodos, 2005; Hodos, 2009). However, the spinal cord does not typically occupy the entire volume of the neural canal and is far from the only structure in this space, which also contains the medial‐most portions of the peripheral nervous system (dorsal and ventral rami, the spinal nerve proper), glial support cells, meninges that support and protect the spinal cord, vasculature that supplies the cord and the vertebrae, structural fat in mammals (Beaujeux et al., 1997; Reina et al., 2009; Sions et al., 2018), and pneumatic diverticula of the respiratory system in birds (Atterholt & Wedel, 2022; Cover, 1953; Müller, 1908; O'Connor, 2006). These varied structures often result in the formation of expansions, cavities, channels, and foramina within the neural canal, many of which are reliable osteological correlates of the soft tissues that produced them (see reviews in Wedel et al., 2021; Atterholt et al., 2024). These correlates may, in turn, be used to infer the presence of their associated soft tissue structures in fossil organisms, providing insights into postcranial neuroanatomy, vascular anatomy, and even respiratory anatomy. Thus, close examination of the neural canal in fossil organisms can yield important new information about the evolution of soft tissue systems and their related physiology.

**FIGURE 1:**
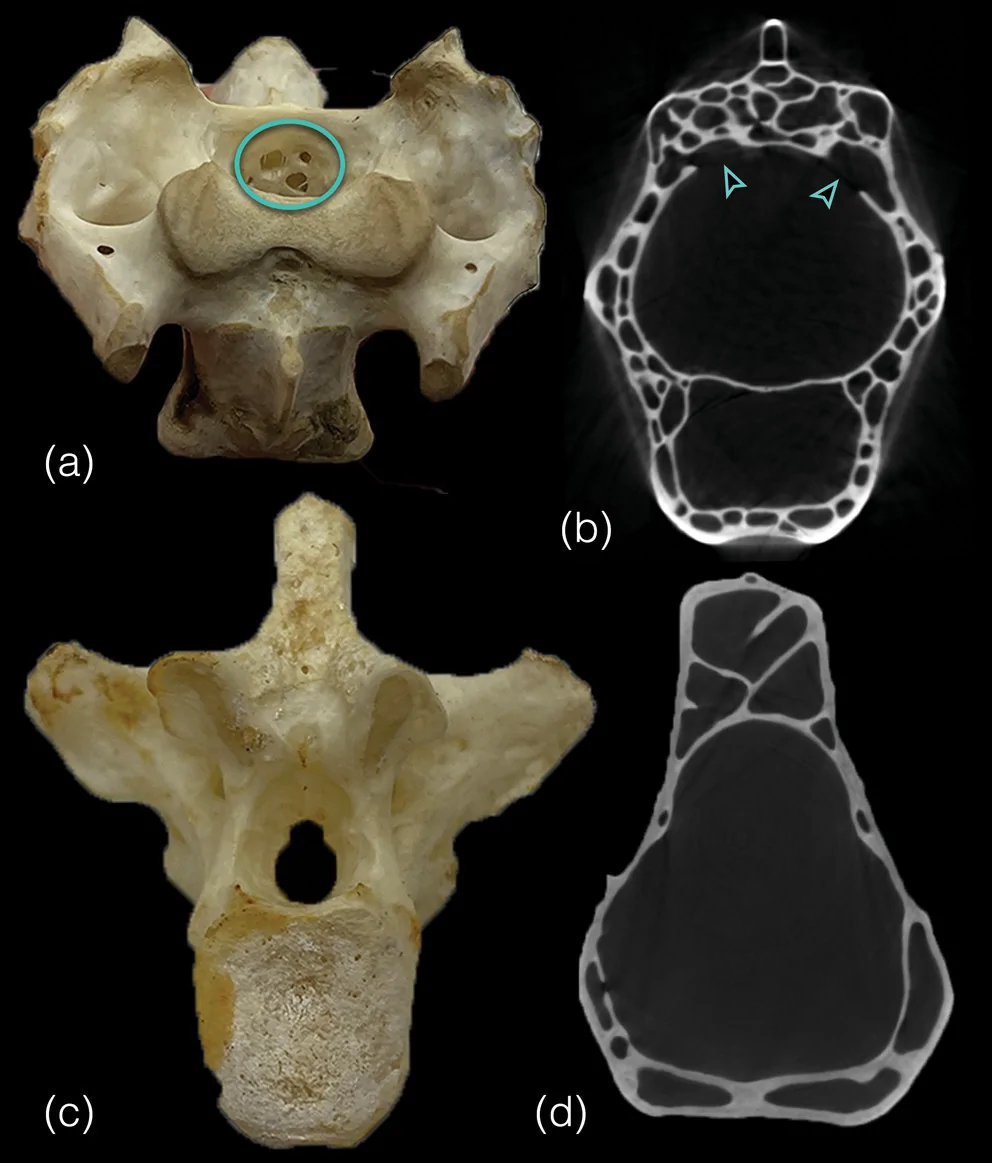
Osteological correlates of paramedullary diverticula (pneumatic foramina in the neural canal) and the internal vertebral sinus (bilobed neural canal geometry) in extant birds as seen in both dry skeletal specimens and CT scans. (a) Wattled crane (*Grus carunculata*, LACM 112175) cervical vertebra with pneumatic foramina (circled) in the roof of the neural canal. (b) Shoebill (*Balaeniceps rex*, LACM 116167) cervical vertebra with pneumatic foramina (arrows) in the roof of the neural canal. (c) Shoebill (*B. rex*, LACM 116167) thoracic vertebra with a bilobed neural canal. (d) Black‐footed albatross (*Phoebastria nigripes*, LACM 115139) cervical vertebra with a bilobed neural canal.

An example of soft tissue structures associated with distinct osteological correlates in the neural canal is paramedullary diverticula (PMDs). In extant birds, these are small diverticula projecting from the air sacs and lungs that sit in contact with the spinal cord and dura mater at intervertebral joints and inside the neural canal (Cover, 1953; Müller, 1908; O'Connor, 2006). In a broad phylogenetically broad survey of these structures in extant birds, Atterholt and Wedel (2022) reported two specific osteological correlates of PMDs: pneumatic foramina inside the neural canal (Figure 1a,b), and a rugose, pocked texture of the bony walls of the neural canal.

Another soft tissue structure in the neural canal that may leave behind osteological traces in birds is the extradural dorsal spinal vein (EDSV) (“internal vertebral sinus” of Baumel, 1993). This is a venous structure located dorsal to the spinal cord and situated between the dura mater and the periosteum (Baumel, 1993; Lob, 1967). A similar and possibly homologous dorsal venous sinus is present in extant crocodilians (Zippel et al., 2003). In both crocodilians and extant birds, the neural canal has a bilobed shape in cross‐section, similar to a figure‐8, in which the spinal cord occupies the ventral “lobe” and the venous structure the dorsal “lobe” (Figure 1c,d). This canal geometry is an osteological correlate of the EDSV (Atterholt et al., 2024). Although this structure appears to be present in multiple extant bird lineages (Columbiformes [Baumel, 1993]; Galliformes [Lob, 1967]; Charadriiformes, Pelecaniformes, Procellariiformes, and Suliformes [personal observation]), its evolutionary history remains uncertain as it has yet to be documented in Mesozoic stem‐group birds. In particular, whether the EDSV was plesiomorphic for Neornithes remains unclear, and evaluating fossil remains of near‐crown stem birds within Ornithurae may reveal the ancestral condition of the bird crown group.

Here, we examined the vertebrae of the Mesozoic ornithurine birds *Ichthyornis dispar* and *Janavis finalidens* using high‐resolution μCT scans of previously described material (Benito, Chen, et al., 2022; Benito, Kuo, et al., 2022) for osteological correlates of PMDs and the EDSV. These taxa constitute the only well‐known representatives of Ichthyornithes and are extremely similar in their overall morphology, yet *Janavis* exhibits a much higher degree of postcranial pneumaticity than *Ichthyornis*, particularly within its axial skeleton, possibly related to its larger body size (Benito, Chen, et al., 2022). We compare the morphology of the neural canals in these Mesozoic birds with those of extant birds and explore the paleobiological implications of the soft tissues inferred to be associated with these osteological correlates.

## 2 MATERIALS AND METHODS

The *I. dispar* specimens examined in this study (Benito, Chen, et al., 2022) originate from the middle‐to‐late Santonian Niobrara Formation of Kansas, USA (KUVP 25472 and 119673) and the early Campanian Mooreville Chalk in Alabama, USA (ALMNH 3316). The holotype of *Janavis* (Benito, Kuo, et al., 2022) is from the late Maastrichtian Valkenburg Member of the Maastricht Formation from a locality in Liège Province, Belgium. The identity and relative position of each examined vertebra within the axial series follow the identifications of Benito, Chen, et al. (2022) and Benito, Kuo, et al. (2022). *Ichthyornis* is considered to have a total presacral count of 21 vertebrae based on multiple specimens, divided into 11 cervical and 10 thoracic vertebrae (Benito, Kuo, et al., 2022). *Janavis* appears to exhibit the same presacral count, based on comparison with *Ichthyornis* (Benito, Chen, et al., 2022). We use a shortened notation for those vertebrae with known identities, with “C” for cervical and “T” for thoracic, followed by the position (i.e., C6 is the 6th cervical, T2 is the 2nd thoracic and the 13th presacral); a slash denotes uncertainty (C8/9 is possibly the 8th or 9th cervical). Thoracic vertebrae 5 to 10 (the 15th to 21st presacrals) are not ordered and are referred to as “indeterminate mid‐thoracic,” as these are extremely similar in morphology and any ordination would be tentative. Similarly, the caudal vertebrae of *Ichthyornis* are unordered as the total caudal vertebral count is unknown. We note that the vertebra of *Janavis* previously identified as C7/8 (Benito, Kuo, et al., 2022) has been reinterpreted here as an indeterminate anterior thoracic, and two previously unimaged indeterminate cervical vertebrae have been identified here as C5 and C8/9 based on new scan data.

In *Ichthyornis* (ALMNH 3316) we examined the axis, five post‐axial cervical vertebrae, five thoracic vertebrae, and the synsacrum; in *Ichthyornis* (KUVP 25472) we examined three cervical, nine thoracic, and three caudal vertebrae; and in *Ichthyornis* (KUVP 119673), we collected data from two cervical, two thoracic, and two indeterminate caudal vertebrae. In *Janavis* (NHMM RD 271) we collected data from three cervical and seven thoracic vertebrae. A summary of observations for these elements can be found in Table 1. We were unable to acquire usable data from all vertebral elements of all specimens due to occasional instances of taphonomic crushing and poor preservation.

**TABLE 1:** Summary of evidence for PMDs and vascular traces in the neural canals of vertebrae of *Ichthyornis* and *Janavis*.

Specimen and element	Pneumatic foramina in canal	Bilobed Canal geometry	Pained ventrolateral channels in canal
*Ichthyornis* (KUVP 25472)			
Cervical 6/7	✓	X	?
Cervical 10	✓	X	X
Cervical 11	✓	X	✓
Thoracic 1 (12th presacral)	?	✓	X
Thoracic 2 (13th presacral)	✓	X	X
Thoracic 4 (15th presacral)	?	✓	X
Indet. Mid‐thoracic 1	✓	✓	?
Indet. Mid‐thoracic 2	X	✓	X
Indet. Mid‐thoracic 3	X	✓	X
Indet. Mid‐thoracic 4	?	✓	X
Indet. Mid‐thoracic 5	✓	✓	X
Indet. Mid‐thoracic 6	?	✓	X
Indet. Caudal 1	?	X	✓
Indet. Caudal 2	X	X	✓
Indet. Caudal 3	?	X	✓
*Ichthyornis* (KUVP 119673)			
Cervical 8/9	✓	?	X
Cervical 11	✓	X	?
Thoracic 1 (12th presacral)	✓	?	X
Thoracic 2 (13th presacral)	✓	?	X
Indet. Caudal 1	✓	X	✓
Indet. Caudal 2	✓	X	✓
*Ichthyornis* (ALMNH 3316)			
Axis	X	X	✓
Cervical 7/8	?	?	X
Thoracic 1 (12th presacral)	?	?	X
Thoracic 2 (13th presacral)	?	?	X
Indet. Mid‐thoracic 1	X	✓	X
Indet. Mid‐thoracic 2	?	✓	X
Sacrals	X	X	✓
*Janavis* (NHMM RD 271)			
Cervical 5	✓	X	?
Cervical 6/7	✓	X	X
Cervical 8/9	✓	X	?
Thoracic 1 (12th presacral)	✓	X	X
Thoracic 2 (13th presacral)	✓	✓	X
Thoracic 3 (14th presacral)	?	?	X
Thoracic 4 (15th presacral)	X	✓	X
Indet. Anterior Thoracic	✓	X	?
Indet. Mid‐thoracic 1	?	✓	X
Indet. Mid‐thoracic 2	?	?	X

The specimens of *Ichthyornis* were μCT scanned at the University of Texas High‐Resolution X‐ray CT Facility using a NSI scanner (scan parameters provided in the Supplementary Information of Benito, Kuo, et al., 2022). *Janavis* was μCT scanned at the Cambridge Biotomography Centre (CBC) using a Nikon 49 Metrology XT H 225 ST high‐resolution CT scanner (scan parameters provided in the Supplementary Information of Benito, Chen, et al., 2022). The region of the *Janavis* holotype main block containing disarticulated cervical and thoracic vertebrae was recently rescanned at the CBC (125 kV, 120 μA, copper filter, source to object 231.53 mm, source to detector 1028.06 mm, 4523 projections, voxel size = 0.0338 mm). Scans were reconstructed and digitally segmented using VGSTUDIOMAX 3.30, 3.40 and 2024.4 (Volume Graphics). CT data for *Ichthyornis* and *Janavis* are available on Morphosource (Project ID: I000405009 and Project ID 000444955, respectively; see Benito, Chen, et al. (2022); Benito, Kuo, et al. (2022) for links and further information). Reported measurements were taken at the greatest diameter or mediolateral width of a given foramen or fossa.

For comparative purposes, CT imaging was also undertaken for vertebrae from several specimens of extant birds from the Ornithology Collections at the Natural History Museum of Los Angeles County. These were scanned at Western University of Health Sciences in Pomona, CA, on a Brukker SkyScan 1275 high‐speed μCT scanner at 100 μA and 70–100 kV, using an aluminum filter or no filter depending on the density of the specimens; voxel sizes ranged from 17 to 50 μm contingent upon the size of individual elements. CT data for these specimens are also available on Morphosource (https://www.morphosource.org/projects/000779811?locale=en).

The three‐dimensionality of the well‐preserved fossil vertebrae lends them to this investigation, and μCT scans of the specimens greatly facilitate the investigation of the neural canals. Data were primarily collected through careful observation of two‐dimensional image stacks of individual elements, rather than from segmented three‐dimensional models. Two‐dimensional images facilitate visualization of the structures under investigation and are especially helpful in identifying foramina and determining whether they are vascular or pneumatic in nature (a methodology previously established, as in Schwarz & Fritsch, 2006; Atterholt & Wedel, 2022; Aureliano et al., 2022).

Institutional Abbreviations: ALMNH, Vertebrate Paleontology Collection, Alabama Museum of Natural History, University of Alabama, Tuscaloosa, AL, USA; KUVP, Vertebrate Paleontology Division, University of Kansas Biodiversity Institute and Natural History Museum, Lawrence, KS, USA; LACM, Natural History Museum of Los Angeles County, Los Angeles, CA, USA; NHMM, Natuurhistorisch Museum Maastricht, Maastricht, The Netherlands.

## 3 RESULTS

### 3.1 Paramedullary diverticula

#### 3.1.1 Ichthyornis

**FIGURE 2:**
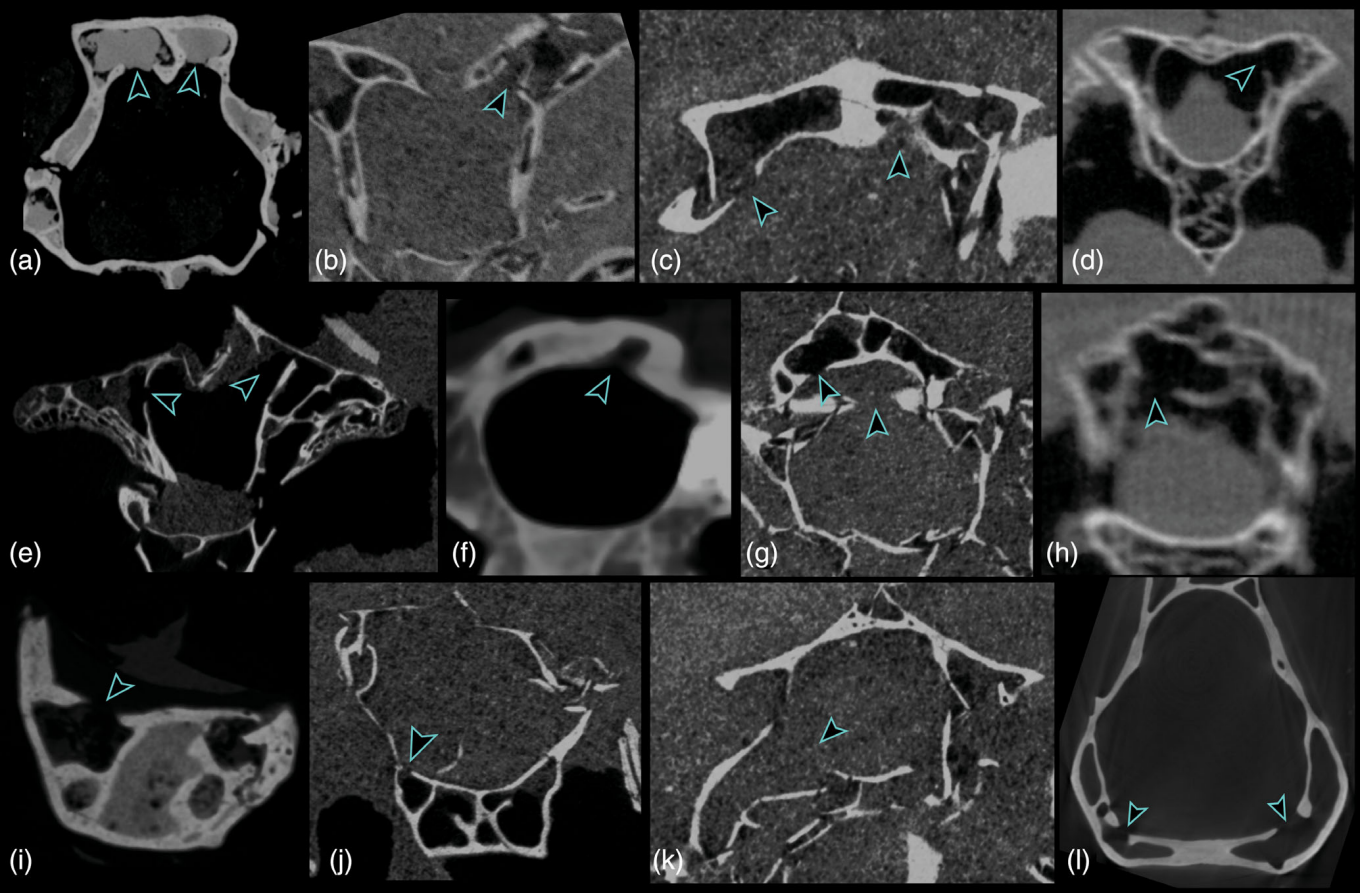
Evidence of paramedullary diverticula in *Ichthyornis dispar* and *Janavis finalidens*, with comparable structures in extant avians: pneumatic foramina (arrows) in the roof of the neural canal through which the posterior zygapophyses are pneumatized (a–d); pneumatic foramina (arrows) in the lateral portions and roof of the neural canal through which the transverse processes (e) and vertebral arch (f–h) are pneumatized; and pneumatic foramina (arrows) in the floor of the neural canal through which the centrum is pneumatized (i–k). (a) *Ichthyornis* (KUVP 25472) C11; here the pneumatic chambers in the arch have been infilled with matrix. (b) *Janavis* (NHMM RD 271) T1. (c) *Janavis* (NHMM RD 271) indeterminate anterior thoracic. (d) Brown skua (*Stercorarius antacticus*) cervical vertebra. (e) *Janavis* (NHMM RD 271) C5. (f) *Ichthyornis* (KUVP 119673) C11. (g) *Janavis* (NHMM RD 271) T1. (h) Brown skua (*S. antacticus*) cervical vertebra. (i) *Ichthyornis* (KUVP 25472) C6/7. (j) *Janavis* (NHMM RD 271) C6/7. (k) *Janavis* (NHMM RD 271) indeterminate anterior thoracic. (l) Black‐footed albatross (*Phoebastria nigripes*, LACM 115139) cervical vertebra.

There is extensive evidence of PMDs in the cervical and thoracic vertebrae of *Ichthyornis* KUVP 25472 (Table 1). C6/7 has pneumatic foramina located in the dorsolateral, lateral, and ventrolateral portions of the canal, which connect with pneumatic chambers in the centrum (Figure 2i), pedicle, and lamina (Figure S1A–D). These range in size from 1.04 to 0.23 mm. C10 has four pneumatic foramina (0.26–0.30 mm) in ventrolateral and lateral areas of the canal through which the transverse processes are pneumatized, as well as what appears to be an elongate and asymmetrical pneumatic fossa in a ventrolateral position of the right wall of the canal (Figure S1E–H). In C11 (Figure 2a) a pair of elongate channels run along the roof of the neural canal and extend for nearly its entire length. These are interpreted as long pneumatic fossae, which iteratively connect to pneumatic spaces within the vertebral arch and the postzygapophyses via several pairs of large pneumatic foramina (0.48–1.00 mm; Figure S1I,J). The neural canals of T1 and T2 (12th and 13th presacrals) were too crushed and distorted to discern whether these channels are also present in these elements. However, at least one clear pneumatic foramen (0.55 mm) is present in T2 (13th presacral), arising dorsally within the canal and becoming a large chamber (1.23 mm) pneumatizing the vertebral arch (Figure S1L–N). Evidence of PMDs is absent or not visible due to crushing in all more caudally positioned vertebral elements (thoracic and free caudal vertebrae), except indeterminate mid‐thoracic vertebra 5 in which there is a single foramen in the floor of the canal (Figure S1T).

In KUVP 119673, pneumatic fossae and foramina are also abundantly present in the neural canals of the two cervical vertebrae and two thoracic vertebrae in which preservational quality permitted data collection (Table 1). The neural arch of C8/9 is too crushed and incomplete to discern pneumatic structures; however, a single, large (1.17 mm) pneumatic foramen is present in the floor of the canal, continuous with an elongate and sinuous pneumatic space that ultimately expands and subsumes neighboring pneumatic cells (Figure S2E,F). C11 (Figure 2f; Figure S2C–F) has five pneumatic foramina in the roof of the canal, through which the arch and both postzygapophyses are iteratively pneumatized. This element exhibits a particularly elaborate arrangement, with several pneumatic foramina that expand greatly and subsequently give rise to additional foramina within walls of the new pneumatic chambers they form (Figure S2C–F). As also seen in the cervicothoracic vertebrae of KUVP 25472, this vertebra has large, paired and elongate pneumatic fossae forming channels in the roof of the canal and giving rise to many of these foramina, although here these fossae do not extend the entire length of the canal but are instead restricted to its posterior region.

T1 (12th presacral) of this specimen is well preserved and clearly shows several pneumatic features, including a large, midline foramen (2.32 mm) extending into the arch. The space continuous with this opening expands to eventually merge with the main portion of the canal (superficially giving a bilobed appearance similar to that described below in association with the dorsal spinal vein), and a second dorsal midline foramen appears in the “new” arch (Figure S2H–J). Posteriorly, there are also foramina via which the postzygapophyses are pneumatized (Figure S2G). T2 (13th presacral) has been subject to asymmetrical crushing, but it is still possible to discern two consecutive left dorsolateral foramina and a left lateral foramen, ranging from 0.30 to 0.80 mm (Figure S2K–N). Ultimately, this element also exhibits pneumatization of both postzygapophyses via foramina inside the canal.

Finally, both of the two preserved free caudal vertebrae have pneumatic foramina extending into the walls of the canal (Figure S2O,Q). These are the only caudal vertebrae in our dataset in which pneumatic foramina were observed inside the canal, although the three caudals from KUVP 25472 are incomplete and missing neural arches, and all caudal vertebrae were highly pneumatized in general.

The neural canals of preserved cervical and thoracic vertebrae of ALMNH 3316 were too crushed and/or incompletely preserved to definitively comment on evidence of PMDs, while the synsacrum of ALMNH 3316 did not exhibit evidence of them. However, because these structures are not always associated with osteological correlates, an absence of evidence is not evidence of their absence.

#### 3.1.2 Janavis

Evidence of PMDs is even more extensive in *Janavis* (NHMM RD 271), which aligns with previous observations that this taxon exhibits a greater degree of vertebral pneumaticity than *Ichthyornis* (Benito, Kuo, et al., 2022). Pneumatic foramina inside the neural canal are present in elements identified as C5, C6/7, C8/9, T‐2 (12th and 13th presacrals), and an anterior indeterminate mid‐thoracic (Table 1).

In C5 a large opening (1.63 mm) is present on the right side, continuous with a pneumatic chamber that expands to fill almost the entire volume of the right transverse process (Figure 2e). Another foramen (0.43 mm) is present dorsolaterally on the left, through which the left transverse process is also pneumatized (Figure S3B,C). A third foramen (1.56 mm) is present at the midline of the roof of the canal, continuous with an elongate pneumatic space into the neural arch and spinous process (Figure S3A).

In C6/7 there are numerous pneumatic foramina present in the neural canal (Figure S3D–G). An opening that initiates on the left side near the anterior end migrates to the roof of the canal and pneumatizes the transverse process and ultimately the arch. Another foramen (0.37 mm) is located on the right side of the canal, while another small (0.25 mm) ventrally positioned foramen pneumatizes the centrum (Figure 2j). Two additional large foramina (0.40 and 0.58 mm, respectively) open into the left ventrolateral part of the canal near its midpoint and posterior end. Finally, a pair of large dorsal fossae are located near the posterior end, which are likely pneumatic and appear to be continuous with pneumatic spaces in the postzygapophyses, although crushing makes it difficult to determine this definitively.

C8/9 has a large pneumatic foramen (0.64 mm) directly into a large chamber in the centrum, located caudally within the neural canal. Near this same portion of the canal, dorsolateral foramina on the right and left walls pneumatize the postzygapophyses (Figure S3H,I). In both C6/7 and C8/9, numerous other possible pneumatic foramina are present, but these could not be unequivocally identified as such due to crushing of the element.

T1 (12th presacral) is also repeatedly and extensively pneumatized from within the neural canal. A large pneumatic foramen (0.44 mm) opens into the arch and spinous process near the anterior end (Figure S3J). At the mid‐point of the canal, a second pneumatic foramen opens into the arch and rapidly inflates into a large pneumatic space, eventually expanding the cavity to the point that it becomes continuous with the neural canal, now increased in size dorsoventrally. The neural arch is subsequently pneumatized again via this space (Figure 2g). Paired dorsal channels are present posteriorly; that on the right is continuous with three pneumatic foramina into the right postzygapophysis, while that on the left is continuous with one pneumatic foramen into the left postzygapophysis (Figure S3K).

In the anterior portion of the neural canal of T2 (13th presacral), there is a large pneumatic foramen (0.75 mm) in the floor of the canal that opens into the centrum (Figure S3M). The ventral foramen closes off quickly, but the dorsal one persists, forming an anteroposteriorly elongate opening. At approximately the midpoint of the canal, a lateral foramen appears, connecting with a small chamber in the right pedicle (Figure S3N).

An indeterminate anterior thoracic also preserves multiple pneumatic foramina. This vertebra is too damaged to clearly establish its absolute position but appears to be an anterior thoracic and part of the cervicothoracic transition. The pre‐zygopophyses are pneumatized via foramina that originate in the dorsal part of the canal (Figure 2c). There is a very large foramen (2.11 mm) on the left side of the canal that extends into the left part of the centrum as a large pneumatic space (Figure 2k); this persists along most of the length of the canal. Three smaller foramina (ranging from 0.22 to 0.34 mm) iteratively pneumatize the arch, two of which are located roughly in the center of the roof of the canal, and one offset to the right.

### 3.2 Extradural dorsal spinal vein

**FIGURE 3:**
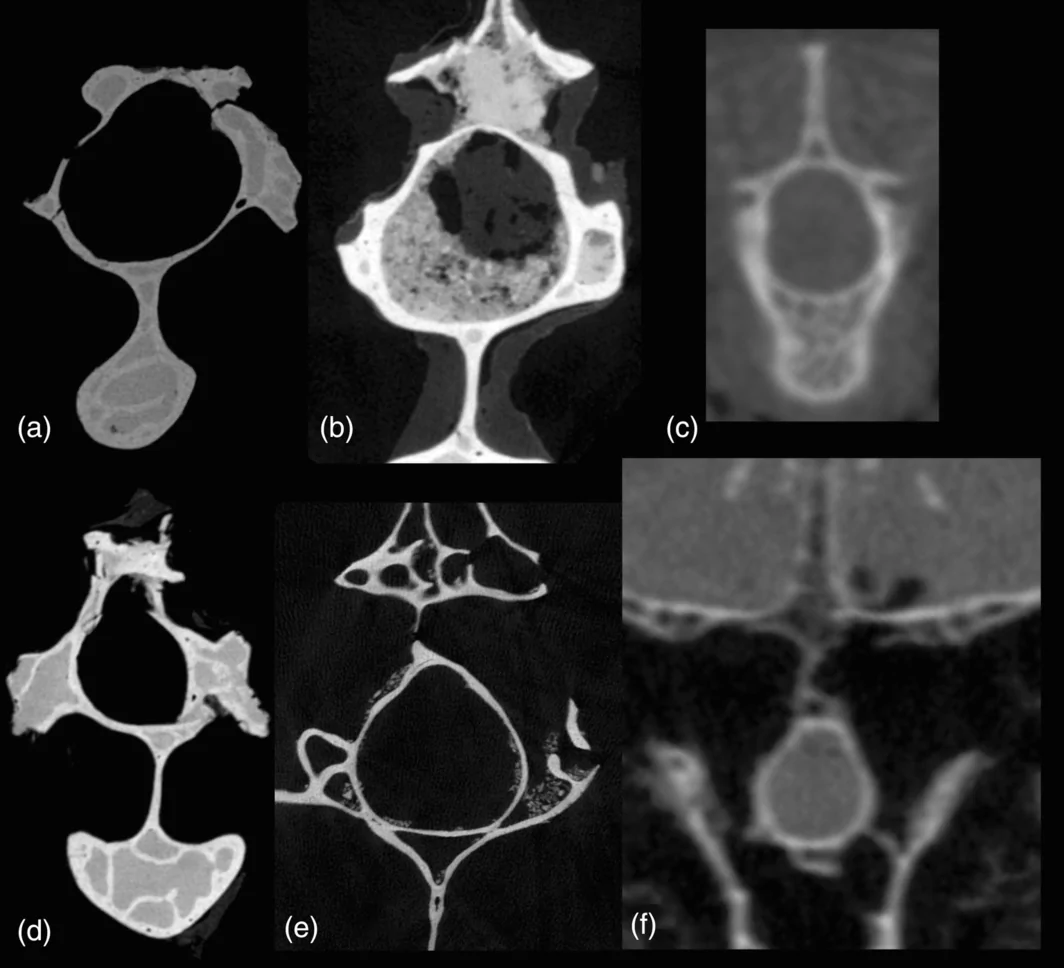
Bilobed neural canals in *Ichthyornis dispar* and *Janavis finalidens* providing evidence of a large, extradural dorsal spinal vein. Comparable anatomy in extant avians also shown. (a) *Ichthyornis* (ALMNH 3316) indeterminate mid‐thoracic vertebra 1. (b) *Ichthyornis* (KUVP 25472) indeterminate mid‐thoracic vertebra 3. (c) Blue petrel (*Halobaena caerulea*) thoracic vertebra. (d) *Ichthyornis* (KUVP 25472) T1. (e) *Janavis* (NHMM RD 271) indeterminate mid‐thoracic vertebra 1. (f) Brown skua (*Stercorarius antacticus*) thoracic vertebra.

T1 (12th presacral), T4 (15th presacral), and indeterminate mid‐thoracic vertebrae 1–6 of *Ichthyornis* (KUVP 25472), and two indeterminate mid‐thoracic vertebrae of *Ichthyornis* (ALMNH 3316) exhibit a distinct bilobed geometry of the vertebral canal in cross‐section (see Figure 3a,b,d for examples). In *Janavis* (NHM RD 271), T2 (13th presacral), T4 (15th presacral), and an indeterminate mid‐thoracic vertebra (Figure 3e) also exhibit this bilobed geometry. In all these vertebrae from both taxa, the ventral “lobe” is larger than the dorsal, though in *Janavis*, this size disparity is greater. Both morphologies are well within the range of variation seen in extant birds (Figure 3), where variation in the relative size of the two portions of such a bipartite neural canal is common among taxa, among individuals within a species, and even among vertebrae of a single individual. Both morphologies are also visible in CT scans of extant birds, where the spinal cord and extradural dorsal spinal vein are visible in situ within a bilobed neural canal (Figure 3c,f). We thus interpret this neural canal shape as evidence for the presence of a large EDSV in *Ichthyornis* and *Janavis*, suggesting that this structure originated prior to the origin of the major clade Ornithurae. This can be distinguished from a superficially similar canal shape sometimes formed by PMDs (as described above in KUVP 119673) by the persistence of the bilobed geometry throughout the entire length of the canal when formed around the vascular structure, rather than limited to only a section as is the case when sculpted by the respiratory structure.

We note that we did not observe evidence of an EDSV in *Ichthyornis* (KUVP 11673). It may seem unusual that two individuals (KUVP 25472 and ALMNH 3316) would have evidence of an EDSV, while a third would not. However, the vertebrae of *Ichthyornis* (KUVP 11673) were either too poorly preserved to discern the geometry of the neural canal or had the dorsal region of the canal substantially reshaped by bone resorption induced by the presence of PMDs. This does not mean such a structure was absent in this individual, as it is entirely possible for the EDSV and PMDs to coexist, although the latter may obscure osteological evidence of the former. The fact that there is clear evidence of an EDSV in the other two *Ichthyornis* specimens, combined with the improbability that such a major vascular structure would be variably present, suggests strongly that this vein ubiquitously characterizes this taxon regardless of a lack of osteological evidence in one individual.

### 3.3 Paired ventrolateral vessels

The osteological correlate described above for the extradural dorsal spinal vein sets a precedent for the potential for the geometry of the neural canal to be shaped by spinal vessels around which the bone develops, in addition to the spinal cord itself. In our survey of fossil material for this study, we sometimes observed the presence of paired channels in the ventrolateral portions of the neural canal. In some cases, these channels bore connections to pneumatic chambers in the centrum, in which case we cannot rule out the possibility that they are elongate fossae sculpted by PMDs. However, some did not connect with any pneumatic spaces, but instead iteratively gave rise to small vascular foramina entering the centrum, supporting the interpretation of these channels as vascular traces. In *Ichthyornis*, such vascular channels are present in the atlas, a posterior cervical vertebra, the synsacrum, and the caudal vertebrae, though the size and consistency of these channels vary greatly among these regions. Similar channels could not be unequivocally identified in any of the vertebrae of *Janavis*, although incomplete preservation and crushing of these elements may obscure such features; additionally, this specimen does not preserve any of the specific vertebrae in which paired ventrolateral channels were observed in *Ichthyornis*.

**FIGURE 4:**
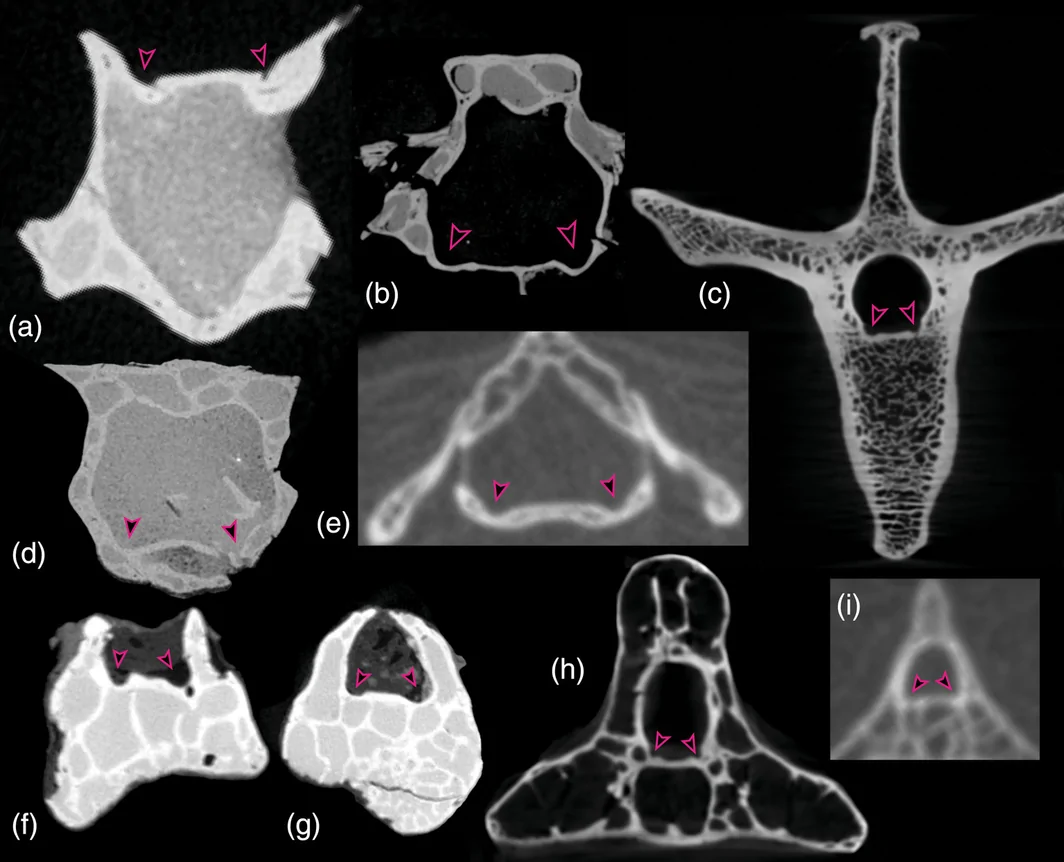
Paired ventrolateral channels *Ichthyornis dispar*, and examples of similar structures in extant avians. (a) *Ichthyornis* (ALMNH 3316) axis; note that the channel on the right has just given rise to a neurovascular foramen. (b) *Ichthyornis* (KUVP 25472) vertebra 11. (c) King penguin (*Aptenodytes patagonicus*, LACM 99854) thoracic vertebra. (d) *Ichthyornis* (ALMNH 3316) sacral vertebra. (e) Blue petrel (*Halobaena caerulea*) sacral vertebra. (f) *Ichthyornis* (KUVP 25472) indeterminate caudal vertebra 1. (g) *Ichthyornis* (KUVP 25472) indeterminate caudal vertebra 2. (h) Common loon (*Gavia immer*, LACM 112761) caudal vertebra. (i) Antarctic prion (*Pachyptila desolata*) caudal vertebra.

In *Ichthyornis*, the most cranial vertebra in which these ventrolateral channels were observed was the axis of ALMNH 3316 (Figure 4a). In this element, these channels are not present throughout the length of the neural canal, but are restricted to the middle portion. They are small (0.23 mm) yet distinct, each of which gives rise to three very small vascular foramina (0.05–0.09 mm) that perforate the centrum. They were also present in C11 of KUVP 25472 (Figure 4b), appearing considerably larger (0.70 mm) than in the axis of ALMNH 3316. They are also in the sacral vertebrae of ALMNH 3316 (Figure 4d), albeit more subtly than in the axis. However, despite their small size, they still give rise to numerous small vascular canals that continue into the centrum.

Relative to the size of the vertebrae, larger ventrolateral vascular channels (ranging from 0.21 to 0.50 mm) are present in three indeterminate caudal vertebrae of KUVP 25472 and two indeterminate caudals of KUVP 119673. In KUVP 119673, they are wide and shallow. In KUVP 25472, they are mediolaterally largest relative to the neural canal and are also the deepest channels into the dorsal surface of the centrum, forming distinct grooves in this area (Figure 4f,g).

The variable size and presence of such channels match what is seen in extant taxa in which we have observed these structures. In the free caudal vertebrae of a common loon (*Gavia immer*, LACM 112761), we observed ventrolateral neurovascular foramina present in all vertebrae, but distinct ventrolateral channels only on one (Figure 4h). In the vertebral column of an emperor penguin (*Aptenodytes forsteri*, LACM 99854), paired ventral channels with vascular foramina are present inconsistently throughout the thoracic vertebral series (Figure 4c), as well as in some cervical and caudal vertebrae.

## 4 DISCUSSION

### 4.1 Paramedullary diverticula

The presence of PMDs in *Janavis* and *Ichthyornis*, both members of the clade Ichthyornithes, is consistent with the strong phylogenetic signal in the presence or absence of these structures in extant birds (Atterholt & Wedel, 2022). Pneumatic structures in *Ichthyornis* and *Janavis* often occur along the length of the neural canal, from the cranial and caudal margins to the midpoint. This implies that, in most of these vertebrae, PMDs extended through the entire vertebral canal and were continuous across at least several consecutive vertebrae (“morphology iii” of Atterholt & Wedel, 2022), a condition seen commonly across a great diversity of extant birds.

*Ichthyornis* shows evidence of PMDs in the cervical, thoracic, and caudal regions of the vertebral column. While rare, this is not unprecedented based on data from extant birds, in which PMDs were observed to persist throughout all regions of the vertebral column in the brown pelican (*Pelecanus occidentalis*) and violet turaco (*Musophaga violacea*) (Atterholt & Wedel, 2022). This strongly implies that PMDs were also present in the sacral region of *Ichthyornis* (and simply did not create any pneumatic fossa or foramina); among extant birds, while PMDs were not always present consistently throughout the complete length of each vertebral region, they were never observed to skip a region entirely (Atterholt & Wedel, 2022).

Pneumatic traces in the vertebrae of *Janavis* indicate that PMDs were present in at least the caudal cervical and cranial thoracic regions. Although T4 does not have evidence of PMDs, it has been observed in extant birds that these diverticula often inconsistently pneumatise the vertebral series, even when present. This pattern of PMDs is consistent with what is known in crown group birds, in which PMDs (when present) are most commonly observed in the cervical and thoracic regions, and become increasingly rare toward the caudal end of the vertebral column (Atterholt & Wedel, 2022). However, the fragmentary nature of the other mid‐thoracic vertebrae, and the absence of more caudal vertebral elements, precludes us from drawing definitive conclusions as to whether PMDs extended into the sacral and caudal regions in *Janavis*.

As noted in Atterholt and Wedel (2022), although PMDs are present in many extant avian taxa, bony traces in the form of foramina or texturing are relatively rare and seem to be the exception rather than the rule. To date, the only taxa in which pneumatic foramina in the neural canal have been observed in multiple vertebrae of a single individual are brown pelicans (Pelecanidae), the black‐footed albatross (Diomedeidae), ostriches (Struthionidae), rheas (Rheidae), and the shoebill (Balaenicipitidae). All of these are large‐bodied birds with hyperpneumatic axial columns (O'Connor, 2004). It is therefore notable that pneumatic foramina in the neural canal are so common in *Ichthyornis* and *Janavis*. Although their presence in *Janavis* might not be unexpected, as it was a large‐bodied bird (~1500 g) that also exhibits hyperpneumatic thoracic vertebral centra, including unique large ventral openings in the anterior thoracics (Benito, Kuo, et al., 2022), *Ichthyornis* had a relatively small body size (~120–480 g) and comparably limited axial pneumaticity (Benito, Chen, et al., 2022). This would seem to suggest that elaborate PMDs may have evolved early in the clade Ichthyornithidae, and that they are characteristic of members of this clade as a whole. Furthermore, while appendicular pneumaticity is common among crown birds, it is notably absent among almost all known avialans outside of the crown group (Field et al., 2025), including *Ichthyornis* (Benito, Chen, et al., 2022). If pneumaticity was truly constrained to the axial skeleton in non‐crown avialans, the numerous and elaborate PMDs observed in Ichthyornithidae may represent a form of hyperpneumaticity that existed within these constraints.

The numerous pneumatic foramina in all regions of the neural canals of many vertebrae in these two taxa, as well as the implied persistence of PMDs through all vertebral regions in *Ichthyornis*, suggests that PMDs in these birds were substantial in size and may even have fully encircled the spinal cord and dorsal spinal vein as documented in the brown pelican, violet turaco, and storks (Atterholt & Wedel, 2022; O'Connor, 2006). Unfortunately, the function of PMDs, if any, remains unknown (Atterholt & Wedel, 2022; Cover, 1953; Müller, 1908; O'Connor, 2006), although their extensive development in some taxa is strongly suggestive of an adaptive function. Atterholt and Wedel (2022) hypothesized they may play a role in cushioning the spinal cord, although this has yet to be tested. The above‐mentioned extant taxa with hyperpneumatic axial skeletons and large, elaborate PMDs all belong to clades that do not have well‐developed notaria (Aires et al., 2022), and it is possible these diverticula are important in cushioning the spinal cord in birds that have more mobile vertebral columns. Ultimately, it is not clear at this time what function extensive PMDs may have served in *Ichthyornis* and *Janavis*.

We also do not yet know when PMDs first appeared in bird‐line archosaurs. Postcranial skeletal pneumaticity is present in pterosaurs, sauropodomorphs, and theropods, but absent in basal dinosauromorphs, ornithischians, and many of the earliest saurischians, implying either parallel gains or losses during the early diversification of Ornithodira (Aureliano et al., 2022; Claessens et al., 2009; O'Connor, 2006). Some kind of air‐sac ventilation of the lungs was probably present in the ancestral dinosauriform, and possibly in the ancestral ornithodiran, but pneumatizing diverticula may have evolved later and in parallel in various ornithodiran lineages (Schachner et al., 2011; Wedel, 2007). Pneumatic fossae or foramina laterally adjacent to the neural canal are widely distributed in pterosaurs, sauropodomorphs, and theropods, implying that pneumatic diverticula were present near the spinal cord from early on in each of those clades (Taylor & Wedel, 2021). Definitive evidence of PMDs has been reported in sauropod dinosaurs, in the form of fossae or foramina in the roof of the neural canal in the brachiosaurid *Giraffatitan* (Schwarz & Fritsch, 2006) and the titanosaurians *Alamosaurus* (Atterholt & Wedel, 2018) and *Ibirania* (Aureliano et al., 2021), but we are not aware of any such reports in pterosaurs or non‐avian theropods. The search for osteological correlates of PMDs and other evolutionary novelties within the neural canals of amniotes is an active frontier of anatomical exploration (see, e.g., Atterholt & Wedel, 2022, Atterholt et al., 2024), and we encourage other researchers to document these features as they find them.

### 4.2 Spinal vasculature

This study also presents the first evidence of specific spinal vascular structures in Mesozoic avialans. The bilobed shape of the neural canals in several vertebrae of both *Ichthyornis* and *Janavis* indicates the presence of an EDSV. This structure is located dorsal to the spinal cord in extant birds, although it has not been described in detail in many taxa, and existing literature reports contradictory information regarding whether these structures are present in all vertebral regions (Baumel, 1993) or restricted to the cervical and thoracic regions (Lob, 1967). Results of the current study indicate that the EDSV was present in at least the cervical and thoracic regions of *Ichthyornis* and *Janavis*. The shape of the neural canal in the sacral and caudal regions (where preserved and visible) is not as clearly bilobed; thus, it is not possible to state whether the EDSV persisted into these caudal regions. However, as is the case with the PMDs, the lack of this osteological correlate does not necessarily imply that the structure was absent in the sacral and caudal vertebrae.

Additionally, the size of the upper “lobe” in the bilobed canals in *Ichthyornis* and *Janavis* suggests that the EDSV was quite large in these taxa. Preliminary research for this study on the neural canals of extant birds indicates that a bilobed canal is often associated with a large internal vertebral sinus, and furthermore that this pair of traits is often seen in oceanic birds. We documented a bilobed neural canal and an EDSV in a brown skua (*Stercorarius antacticus*, Figure 3c), blue petrel (*Halobaena caerulea*, Figure 3f), and Brandt's cormorant (*Phalacrocorax penicillatus*), although they are also present in an eclectus parrot (*Eclectus roratus*). In contrast, we observed the neural canal to be closer to circular in shape (and not bilobed) in birds with a relatively smaller EDSV; these included the song thrush (*Turdus philomelos*), ostrich (*Struthio camelus*), Eastern moa (*Emeus crassus*), emperor penguin (*A. forsteri*), and Anna's hummingbird (*Calypte anna*). These are incidental observations made in the process of collecting comparative data for this study, and we did not conduct a thorough survey of the EDSV or bilobed neural canals across crown group birds. Further research describing these structures in extant birds is necessary to parse out potential phylogenetic and functional correlations.

The consistent presence of ventrolateral, paired channels from which smaller vascular channels repeatedly arise in several vertebrae of *Ichthyornis* suggests that a pair of extradural vessels was present ventrolaterally in the neural canal. There is a paucity of information on spinal vasculature in extant birds, and we could find no source that describes large, extradural, ventrolaterally located, paired vessels. Observations of both CT scan data and dry skeletal material of extant birds confirm the presence of similar bony channels in at least some extant taxa (Figure 4c,h,i). The extant avian specimens that were μCT‐scanned for this study were not contrast‐stained for soft tissue visualization; thus, it is not possible to see specific structures associated with these channels (unlike the EDSV, which is often large enough to be discerned as a distinct structure). However, in both types of data, we consistently observed vascular foramina closely associated with these channels, as in *Ichthyornis*. Notably, somewhat larger paired vascular channels are present in the vertebrae of some extant squamates, including *Varanus komodoensis* and many snakes (Zippel et al., 2001, Figure 3; and pers. obs.), and are known to house a pair of large, extradural veins (Zippel et al., 2001). We suggest that these channels are similarly occupied by a pair of extradural spinal veins in birds, and that the small vascular foramina arising from them are basivertebral veins that would have drained into these spinal veins. Together with the evidence of a large dorsal vein, these observations indicate that an elaborate spinal venous plexus was likely present in *Ichthyornis*, superficially similar to the plexuses observed in snakes (Zippel et al., 2001), crocodylians (O'Connor, 2006), and mammals (Crock & Yoshizawa, 1977). Future studies in extant Aves will be necessary to more thoroughly elucidate variation in spinal vasculature in modern birds. We also postulate that, despite the diminishing size of the spinal cord, the relatively large ventrolateral vessels in the caudal vertebrae of *Ichthyornis* (as implied by the large channels) may have contributed to the blood supply of the rectricial apparatus (see Baumel, 1988).

**FIGURE 5:**
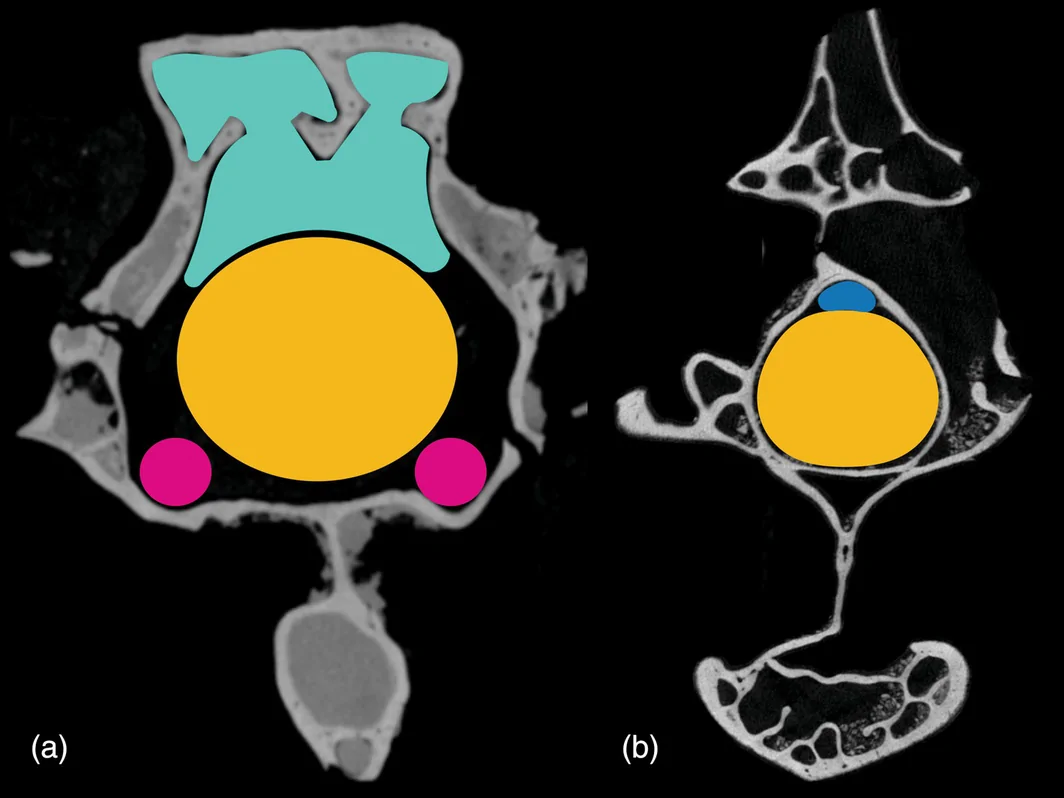
Hypothesized reconstructions of respiratory, vascular, and neurological structures in the neural canals of *Ichthyornis dispar* and *Janavis finalidens*. (a) *Ichthyornis* (KUVP 25472) cervical 11 showing likely arrangement of paramedullary diverticula (green) and paired extradural ventral spinal vessels (pink) relative to the spinal cord (yellow). (b) *Janavis* (NHMM RD 271) indeterminate mid‐thoracic vertebra 1 showing likely arrangement of the extradural dorsal spinal vein (blue) relative to the spinal cord (yellow).

The adaptive or exaptive functions of extradural vasculature in birds are also not well understood. In snakes, the large venous sinus in the neural canal provides an important route for cephalic vascular drainage. The vertebral venous sinus is propped open by the walls of the neural canal and remains patent, and it is therefore particularly important in postures in which the head is elevated where the jugular veins may collapse from insufficient pressure (Zippel et al., 2001). In crocodilians, a large dorsal venous sinus is thought to facilitate variable hemodynamic shunting during diving or thermoregulatory basking (Zippel et al., 2003). In diving seals, posterior blood flow in the vertebral venous sinuses sends blood to the hepatic portal system, helping to push relatively oxygen‐rich blood from expanded hepatic sinuses forward to the heart and ultimately to the brain (Harrison & Tomlinson, 1956; Ronald et al., 1977; see review in Zippel et al., 2003). No extant diving birds swim as deep or stay submerged as long as seals do (Kooyman & Ponganis, 1998), but emperor penguins have been recorded diving nearly 550 m and staying submerged for over 15 minutes at depths of 50 m or less (Kooyman & Kooyman, 1995). Although more research is required to explore this possibility, large vertebral venous sinuses may facilitate that behavior in extant diving birds, and the same may have been true in the seabird‐like Mesozoic taxa *Ichthyornis* and *Janavis*. However, despite being clearly capable of foot‐propelled swimming, both the sternum and hindlimb morphology of *Ichthyornis* suggest that it was not particularly adapted for diving and was more likely to have swum at the surface (Benito, Chen, et al., 2022; Lowi‐Merri et al., 2023). In addition, intraskeletal pneumatic spaces and PMDs would have been incapable of collapsing, and the buoyancy provided by these air reservoirs would have hampered deep dives in *Ichthyornis* and *Janavis* (Figure 5).

### 4.3 Pneumatic versus vascular foramina

In the process of data collection for this study, it was essential to be able to differentiate pneumatic from the many vascular foramina present in the cortical wall of the neural canal. The following is a list of features that frequently characterize pneumatic and vascular foramina respectively (summarized in Table 2). Pneumatic foramina are often quite large and irregularly shaped; vascular foramina are often relatively small and circular or elliptical. Pneumatic foramina usually have a diameter several times greater than the thickness of the bony wall that they perforate; vascular foramina usually have a diameter equal to or less than the thickness of the bony wall that they perforate. Pneumatic foramina are essentially always continuous with large pneumatic spaces inside a bone; large vascular channels will frequently give rise to smaller vascular canals. Pneumatic foramina tend to be highly variable in location and size on a single vertebra (sometimes occurring asymmetrically), between consecutive vertebrae, and among individual organisms; vascular foramina tend to be consistently present in approximately the same location in consecutive vertebrae and among individual organisms of the same species (but see Taylor & Wedel, 2021).

**TABLE 2:** Characteristics helpful for distinguishing pneumatic and vascular foramina on vertebrae.

Pneumatic foramina	Neurovascular foramina
Relatively large, irregular in shape	Relatively small, circular or elliptical in shape
Diameter several times greater than the bony wall they perforate	Diameter less than or equal to the bony wall they perforate
Connect to pneumatic chambers	Give rise to smaller vascular canals
Highly variable in size and location between consecutive vertebrae	Consistent in location and size between consecutive vertebrae

It is essential to acknowledge that variation exists, and these differences are not absolute. In this very paper, we have shown examples of relatively small pneumatic foramina and pneumatic foramina present serially in consecutive vertebrae. When determining whether an opening is vascular or pneumatic, as many of these features as possible should be used in concert. Furthermore, the ability to visualize the internal architecture of a bone (either with CT data or because it is broken) can be very helpful in characterizing a foramen and determining the type of soft tissue structure it is associated with. Ultimately, these criteria are a starting point, and we expect they will be refined and expanded in future investigations.

## 5 CONCLUSION

This study presents the first evidence of PMDs in Mesozoic birds, and provides new information on vascular structures inside the avian neural canal. Our observations indicate that the evolutionary origins of specific structures of the respiratory and vascular systems known to characterize crown‐group birds predate the phylogenetic divergence of the clade Ichthyornithes. Evidence shows that *Ichthyornis* and *Janavis* both had a large and extensive system of PMDs, and that an elaborate extradural spinal venous system was present in *Ichthyornis*. This research also highlights how little is currently known about the anatomy and function of these structures in extant birds. There is a great need for studies further documenting PMDs and parsing out their function(s), and for studies describing spinal vasculature in a greater phylogenetic diversity of avian taxa.

## AUTHOR CONTRIBUTIONS

**Jessie Atterholt:** Conceptualization; investigation; writing – original draft; methodology; writing – review and editing; visualization; validation; software; formal analysis; project administration; data curation; supervision; resources. **M. Grace Burton:** Investigation; writing – review and editing; methodology; validation; software; formal analysis; data curation; resources. **Mathew J. Wedel:** Conceptualization; investigation; writing – original draft; methodology; validation; writing – review and editing. **Juan Benito:** Writing – review and editing; methodology; software; formal analysis; resources; data curation. **Ellen Fricano:** Writing – review and editing; software; formal analysis; methodology. **Daniel J. Field:** Funding acquisition; writing – review and editing; resources.

## Supporting information


**Figure S1.** DICOM slices of various elements of KUVP 25472 showing pneumatic foramina and fossae (green arrows), and bilobed geometry of the neural canal (images without arrows). All images show different foramina and fossae, except for those in white boxes, which are serial images, shown to better display the formation of pneumatic excavations. (A–D) Cervical 6/7. (E–H) Cervical 10. (I–J) Cervical 11. (K) Thoracic 1 (12th presacral). (L–N) Thoracic 2 (13th presacral). (O, P) Indeterminate mid‐thoracic 1. (Q) Indeterminate mid‐thoracic 2. (R) Indeterminate mid‐thoracic 3. (S) Indeterminate mid‐thoracic 4. (T, U) Indeterminate mid‐thoracic 5.

**Figure S2.** DICOM slices of various elements of KUVP 119672 showing pneumatic foramina and fossae (green arrows) and ventrolateral vascular channels (pink arrows) in the neural canal. All images show different foramina and fossae, except for those in white boxes, which are serial images, shown to better display the formation of pneumatic excavations. (A, B) C4. (C–F) C11. (G–J) Thoracic 1 (12th presacral). (K–N) Thoracic 2 (13th presacral). (O, P) Indeterminate caudal 1. (Q, R) Indeterminate caudal 2.

**Figure S3.** DICOM slices of various elements of KUVP 25472 showing pneumatic foramina and fossae (green arrows), and bilobed geometry of the neural canal (images without arrows). All images show different foramina and fossae, except for those in white boxes, which are serial images, shown to better display the formation of pneumatic excavations. (A–C) C5. (D–G) C6/7. (H, I) C8/9. (J, K) T1. (L–N) T2. (O) T4.
